# Evaluating the ready biodegradability of two poorly water-soluble substances: comparative approach of bioavailability improvement methods (BIMs)

**DOI:** 10.1007/s11356-016-6899-3

**Published:** 2016-05-28

**Authors:** Cyril Sweetlove, Jean-Charles Chenèble, Yves Barthel, Marc Boualam, Jacques L’Haridon, Gérald Thouand

**Affiliations:** 1 L’Oréal Research & Innovation, Environmental Research Department, 93600 Aulnay-sous-Bois, France; 2 UMR CNRS 6144 GEPEA CBAC Lab, University of Nantes, 85035 La Roche-sur-Yon, France; 3 Eurofins Expertises Environnementales, Ecotoxicology Lab, 54521 Maxéville, France

**Keywords:** Biodegradability, Poorly water-soluble substances, Ready biodegradability test, Bioavailability improvement method, Evaluation strategy, Silicon oil, Silica gel

## Abstract

Difficulties encountered in estimating the biodegradation of poorly water-soluble substances are often linked to their limited bioavailability to microorganisms. Many original bioavailability improvement methods (BIMs) have been described, but no global approach was proposed for a standardized comparison of these. The latter would be a valuable tool as part of a wider strategy for evaluating poorly water-soluble substances. The purpose of this study was to define an evaluation strategy following the assessment of different BIMs adapted to poorly water-soluble substances with ready biodegradability tests. The study was performed with two poorly water-soluble chemicals—a solid, anthraquinone, and a liquid, isodecyl neopentanoate—and five BIMs were compared to the direct addition method (reference method), i.e., (i) ultrasonic dispersion, (ii) adsorption onto silica gel, (iii) dispersion using an emulsifier, (iv) dispersion with silicone oil, and (v) dispersion with emulsifier and silicone oil. A two-phase evaluation strategy of solid and liquid chemicals was developed involving the selection of the most relevant BIMs for enhancing the biodegradability of tested substances. A description is given of a BIM classification ratio (*R*_BIM_), which enables a comparison to be made between the different test chemical sample preparation methods used in the various tests. Thereby, using this comparison, the BIMs giving rise to the greatest biodegradability were ultrasonic dispersion and dispersion with silicone oil or with silicone oil and emulsifier for the tested solid chemical, adsorption onto silica gel, and ultrasonic dispersion for the liquid one.

**Electronic supplementary material:**

The online version of this article (doi:10.1007/s11356-016-6899-3) contains supplementary material, which is available to authorized users.

## Introduction

Chemicals produced by human activities are a potential source of environmental pollution, and concerns regarding their potential to cause adverse effects are greater when they are considered to be persistent. In many cases, degradation by bacteria, i.e., biodegradation, is a major factor in the assessment of the environmental persistence of chemicals (Ramade 1992). Interest in biodegradability testing dates back to 50 years (Borstlap and Kooijman 1963). Today, biodegradability is a key parameter in several international regulations on chemical substances, such as the European Union regulation REACH (ECHA 2006) and the European CLP regulation (European parliament 2008). In order to evaluate the complete mineralization of chemicals, i.e., ultimate biodegradation, tests have been designed under the umbrella of international organizations such as the Organization for Economic Co-operation and Development (OECD) and International Organization for Standardization (ISO).

Among these tests, the ready biodegradability tests enable the ultimate biodegradability in aqueous media to be assessed under aerobic conditions. Ready biodegradability test highlights the rapid biodegradation of chemicals under most environmental conditions (ECHA 2014).

Difficulties encountered in estimating the biodegradability of poorly water-soluble substances are often linked to their limited bioavailability to microorganisms (Stucki and Alexander 1987; Alexander 1999). Working on improving test solution preparations for poorly water-soluble substances is a potentially interesting approach, since a genuinely realistic approach—i.e., performing tests at the likely environmentally relevant chemical concentration of a few parts per billion (10^−9^ g/g)—is experimentally infeasible without the use of radiolabeled material. Many scientists have worked on this research subject (Gerike 1984; Blok et al. 1985; Thomas et al. 1986; De Morsier et al. 1987; Nyholm 1990; Ramade 1995; Painter 1995 ; Handley et al. 2002; Ingerslev et al. 2000; Dumont et al. 2006; Van Ginkel et al. 2008; Li and Chen 2009; Rodrigues et al. 2013). The most interesting test solution preparation methods described are sonication, adsorption onto solid supports (Nyholm 1990; Ramade 1995; Handley et al. 2002; Li and Chen 2009; Rodrigues et al. 2013), dispersion with emulsifier or solvent, dispersion with silicone oil (Dumont et al. 2006; Van Ginkel et al. 2008), or dispersion with emulsifier and silicone oil. The most appropriate methods for conducting these tests seem to be respirometric methods, which measure CO_2_ production or O_2_ consumption with constant flask stirring (Gerike 1984; Blok et al. 1985; Thomas et al. 1986; OECD 1992).

The main difficulty in comparing or reproducing these different bioavailability improvement methods (BIMs) is the variability of the microbial inoculum used to perform the biodegradability testing (Thouand et al. 1995, 1996; Van Ginkel et al. 1995; Goodhead et al. 2013; Kowalczyk et al. 2015). This variability can even be considered as a factor, making tests conducted with different inocula hardly comparable (Blok and Booy 1984). For example, Sweetlove et al. (2013) showed that ultrasonic dispersion improved the biodegradability of anthraquinone, while Nyholm (1990) concluded the opposite.

Many original bioavailability improvement methods (BIMs) have been described, but no global approach for a standardized comparison of these exists. The latter would be a valuable tool as part of a wider strategy for evaluating poorly water-soluble substances. The purpose of this study was to define an evaluation strategy by assessing five different BIMs on two reference poorly water-soluble substances with ready biodegradability tests.

The choice and performance of BIMs can be influenced by the physical state of the test material. Therefore, one solid and one liquid chemical were selected for evaluation. Anthraquinone was chosen because it is a reference standard in biodegradability tests for poorly water-soluble chemicals according to ISO 10634 (ISO 1995) and European Chemical Industry Council report No. MCC/007 (Comber and Holt 2010). Based on the supplier’s Material Data Safety Sheet (MSDS), it is readily biodegradable (62 % biodegradation at day 28, according to EC Test Guideline C.4.E.) but does not meet the 10-day window (dw) criterion. Isodecyl neopentanoate, used as an emollient, is the only liquid cosmetic ingredient that meets all of the desired criteria; it has a defined chemical structure, is of very high purity and non-toxic to bacteria, and has a low water solubility, an oily form and moderate biodegradability according to the supplier’s MSDS (35.4 % at 28 days in accordance with OECD Test Guideline 301B). This branched-chain ester is predicted readily biodegradable (92 % at 28 days with no stable metabolite) according to the OASIS CATALOGIC Kinetic 301B model, v02.09 (Dimitrov et al. 2011a, 2011b), cited in the REACH guidance (ECHA 2014).

The following BIMs were compared: (i) ultrasonic dispersion, (ii) dispersion using an emulsifier, (iii) adsorption onto silica gel, (iv) dispersion with silicone oil, and (v) dispersion with an emulsifier and silicone oil. The calculation of a BIM classification index is proposed, which enables the different operating conditions to being ranked.

## Materials and methods

### Materials

Anthraquinone (ref.: CAS RN: 84-65-1, 97 % purity, A90004 from Sigma-Aldrich, Saint-Quentin-Fallavier, France) and isodecyl neopentanoate (CAS RN: 60209-82-7, DUB VCI 10 from Stearinerie Dubois, Boulogne-Billancourt, France) were the two reference test substances. Sodium acetate (CAS RN: 127-09-3, Sigma-Aldrich) was used as a positive control substance. Silica gel (CAS RN: 112926-00-8, size 15 μm, Sigma-Aldrich) was chosen according to ISO 10634 (ISO 1995).

Silicone oil AR 20® (CAS RN: 63148-58-3, Sigma-Aldrich) was selected, based on Van Ginkel’s research (Van Ginkel et al. 2008). The emulsifiers chosen among those proposed by the ISO 10634 guideline (ISO 1995) were Synperonic PE/P94® and PE/P103® (formerly Pluronic PE9400® and PE10300®, respectively). Pluronic PE9400® was obtained from BASF SE (Levallois-Perret, France).

Ultrasound treatments—35 kHz at 22 °C—were performed with an Ultrasonic Bath 9L SHE10000 (LaboModerne, Paris, France). Emulsions were made with an IKA® Eurostar Power Control Visc Euro-ST PCVS1 Mixer Stirrer at 50–2000 rpm. Carbon measurements were performed with a CHNS Elementar (VARIO model: Vario El Cube).

The mineral medium used in the ready biodegradability tests was made up according to OECD Test Guideline 301B and contained the following nutrients per liter of ultra-pure water: 85 mg KH_2_PO_4_, 217.5 mg K_2_HPO_4_, 334 mg Na_2_HPO_4_·2H_2_O, 5 mg NH_4_Cl, 27.5 mg CaCl_2_, 22.5 mg MgSO_4_·7H_2_O, and 0.25 mg FeCl_3_·6H_2_O (OECD 1992).

### Biodegradation tests

Two types of biodegradation test, based on CO_2_ measurements, were carried out in this study. A respirometer was used to perform screening tests for BIM selection, followed by a standardized ready biodegradability tests to confirm the results. In order to be considered readily biodegradable according to OECD Test Guidelines (OECD 1992), a biodegradation threshold of 60 % ThCO_2_ has to be reached in a 10-10-dw within the 28-day test period. The 10-dw begins when biodegradation reaches 10 %.

A Respicond VI® respirometer from A. Nordgren Innovations AB (Bygdeå, Sweden) was chosen to compare the different BIMs. Respicond VI® has several advantages: (i) 95 closed test flasks (volume 150 mL) running in parallel allow a high number of operating conditions to being compared in the same test with the same microbial inoculum, (ii) stirring of the test solutions, and (iii) simple and accurate CO_2_ detection to measure biodegradation of the test substance. CO_2_ release was measured as described by Nordgren 1988, based on the change in conductivity of a solution of potassium hydroxide containing trapped CO_2_. The CO_2_ absorbed in this hydroxide solution briefly forms carbonate ions that reduce the solution’s conductivity. This change in conductivity can be calibrated against the amount of absorbed CO_2_ and provides an integrated measurement of respiration. This principle is similar to the one recommended in OECD Test Guideline 301 B (OECD 1992).

OECD Test Guideline 301 B was followed to perform standard tests with two flasks containing the test substance (with or without a BIM) and the inoculum, two flasks containing only the inoculum (with or without a BIM), and one flask containing the reference compound and the inoculum.

Activated sludge was collected from a predominantly domestic sewage treatment plant (Maxéville, France, 500,000 population equivalents). The activated sludge was kept at 20 °C and used within 24 h after collection as described in OECD Test Guideline 301 (OECD 1992). The final dry matter concentration of inoculum used in the Respicond® and standard test flasks was 29.5 ± 0.5 mg L^−1^.

Immediately prior to the start of the Respicond test, 1.5 mL of the microbial inoculum described above (2.95 g of dry matter L^−1^) was added to each flask.

Operating conditions for the standard 301 B tests were the same as those used for the screening tests carried out with the Respicond VI®, except that the amounts were adjusted to 1 L of test solution instead of 150 mL.

Positive controls were prepared with sodium acetate at 20 mg of carbon L^−1^ as the reference substance. Blanks were prepared with the microbial inoculum only. All tests were performed at 21.0 ± 0.2 °C.

### Operating conditions

Each BIM was compared to the reference method result (direct addition of the tested substance to the inoculum) of the same test to avoid inherent variability due to the inoculum, which was sampled in a wastewater treatment plant (Nyholm 1990; Van Ginkel et al. 1995; Thouand et al. 1996; Goodhead et al. 2013).

For the screening tests, the direct addition method was performed by adding 3.7 mg of anthraquinone or 4.0 mg of isodecyl neopentanoate to 148.5 mL of mineral medium in order to obtain a final concentration of 20 mg carbon per liter.

Ultrasonic dispersion was conducted in the same way as direct addition, except that the solution was dispersed for 10 min with the ultrasonic bath at 35 kHz immediately prior to adding the inoculum.

As described in the NF EN ISO 10634 standard (ISO 1995), 150 mg of anthraquinone was adsorbed onto 30 g of silica gel with 150 mg of acetone. The amount of anthraquinone adsorbed onto the silica gel was measured three times with a solid total organic carbon analyzer; the concentration of anthraquinone was 4 mg carbon per gram of silica gel. Thus, 750 mg of silica gel with adsorbed anthraquinone was added to 148.5 mL of mineral medium in each test flask.

In order to adsorb isodecyl neopentanoate onto silica gel, 750 mg of silica gel was mixed for 5 min with 4.0 mg of isodecyl neopentanoate. This mixture was added under magnetic stirring at 250 rpm to 147 mL of mineral medium. Another test solution was prepared with 8.0 mg of silica gel.

An emulsion was made with an emulsifier by mixing 50 mL of Pluronic 9400® at 1 g L^−1^ into a mineral medium with 124 mg of anthraquinone or 134 mg of isodecyl neopentanoate, using an IKA® mixer at 500 rpm for 10 min at room temperature. Of this mixture, 1.5 mL was homogenized with 147 mL of mineral medium.

A silicone oil dispersion was prepared in an Eppendorf® tube by mixing 50 mL of silicone oil with 124 mg of anthraquinone or 134 mg of isodecyl neopentanoate. Of this mixture, 1.5 mL was homogenized with 147 mL of mineral medium to obtain a silicone oil dispersion with a final concentration of 10 mL L^−1^ and 150 μL of this mixture was homogenized with 148.35 mL of mineral medium to obtain a silicone oil dispersion with a final concentration of 1 mL L^−1^.

The mixture of Pluronic PE9400® and silicone oil was prepared with 50 mL of Pluronic 9400® at 1 g L^−1^ in mineral medium and 124 mg of anthraquinone or 134 mg of isodecyl neopentanoate. Of this mixture, 1.5 mL was homogenized with 145.5 mL of mineral medium and 1.5 mL of silicone oil.

The operating conditions are summarized in the Table 1.

**Table 1:** Bioavailability improvement methods used, with their type and mode of action

BIM	Type of action	Mode of action
Ultrasonic dispersion	Physical dispersion	Ultrasonic dispersion
Silicon oil	Chemical emulsion	Emulsion with mechanical dispersion
Surfactant	Chemical emulsion	Emulsion with mechanical dispersion
Surfactant + silicon oil	Chemical emulsion	Emulsion with mechanical dispersion
Silica gel	Physical dispersion	Inert solid support with magnetic stirring dispersion

## Exploitation of results

### Statistical analysis

For screening tests with Respicond VI®, five replicates per test solution and control, and ten replicates per blank, were confirmed to be statistically adequate for experimental biodegradation tests. The median value of the five replicates was calculated for each day and was chosen as the biodegradation value because it is more robust than the mean value of outliers (Falissard 2005). Consequently, it was not appropriate to determine the standard deviation for the Respicond biodegradation results. Therefore, all test results (Respicond with five replicates and standard with two replicates) were evaluated with the median value of the replicates and the median absolute deviation—MAD (1)—as the variability indicator (Wonnacott and Wonnacott 1995), which is less affected by extreme values for results with few replicates.

1 $$ \mathrm{MAD}=\mathrm{median}\left(\left|{x}_i-\tilde{x}\right|\right) $$

with

*x*_*i*_: Result value of the group
*x̃*: Median value of the group

The validity criteria were the same as described in Test Guideline OECD 301 B (OECD 1992), but the use of five replicates instead of two gave rise to an additional validity criterion. The difference between the median value and each replicate value was calculated. The validity criterion was defined by this difference, which was not to exceed 15 % biodegradation for three specific days: the beginning of the 10-dw, the end of the 10-dw, and the end of the test (D28). When at least four out of five replicate values complied with this validity criterion, the test was considered valid.

### Determination of a BIM classification index

**Fig. 1:**
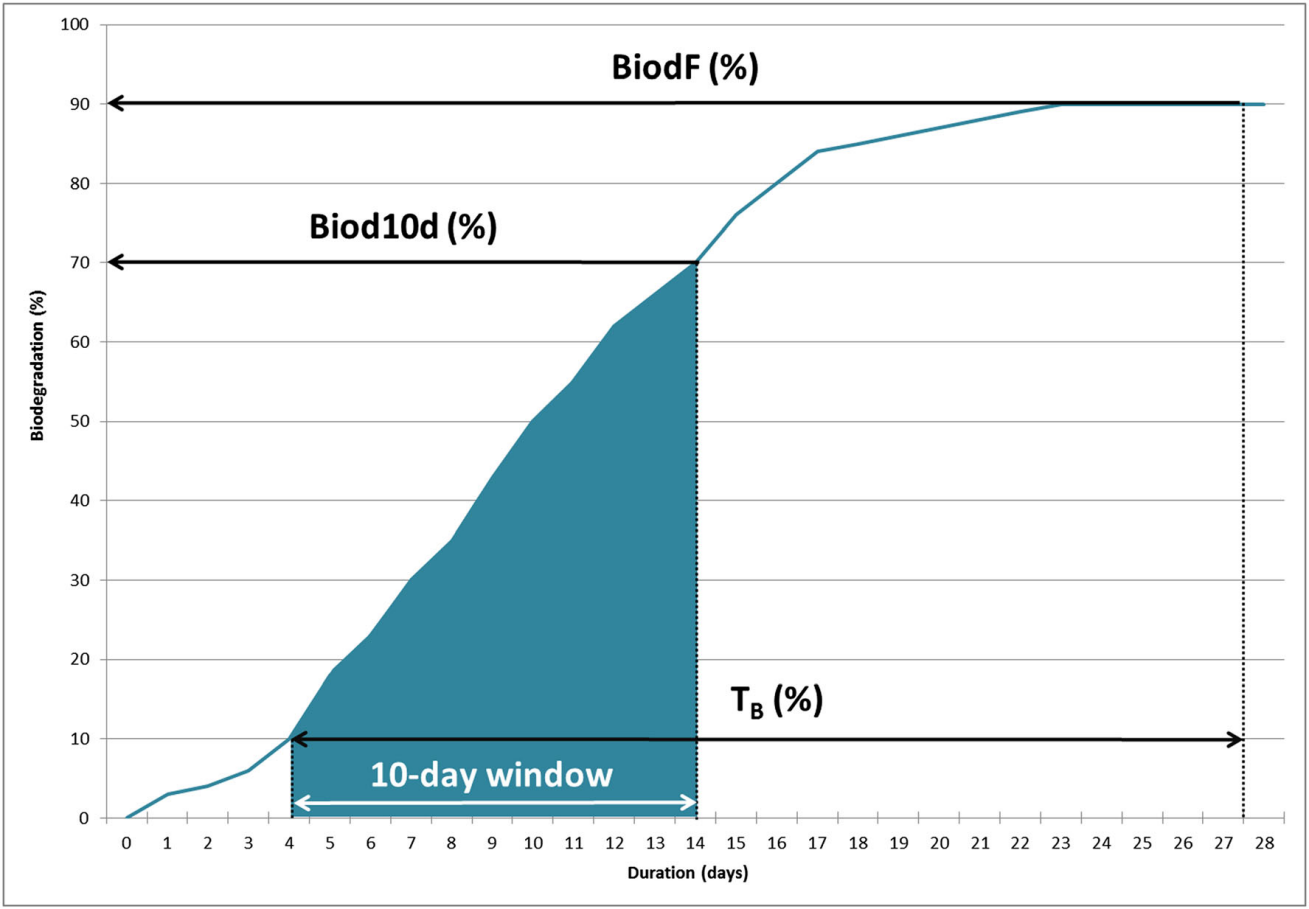
Example of ready biodegradation test curve with parameters *T*_B_, Biod10d, and BiodF identified

OECD biodegradability test results are usually described by two main parameters (Fig. 1): (i) the biodegradation percentage at the end of the 10-dw (Biod10d) and (ii) the biodegradation percentage at the end of the test at day 28 (BiodF). A BIM was considered favorable when the 10-dw began earlier in comparison with direct addition during the 28 days of testing. We calculated a third parameter (iii), the percentage of time available for the bacteria to biodegrade the product over 28 days (*T*_B_) (2).

2 $$ {T}_{\mathrm{B}}=\frac{\left(28-\mathrm{F}\mathrm{D}\right)}{28}\times 100 $$

with

FD: First day when 10 % biodegradation is reached

We considered that these three parameters had the same weight in quantifying the progression of biodegradation during the 28 days of the test. We defined a quantification coefficient, *C*_BIM_ (3), which allows the results obtained using the different BIMs to being compared (Fig. 1).

3 $$ {C}_{\mathrm{BIM}}=\frac{\left({T}_B+\mathrm{Biod}10\mathrm{d}+\mathrm{Biod}\mathrm{F}\right)}{3} $$

with

*T*_B_: Percentage of time available for biodegradation
Biod10d: Percentage of biodegradation at the end of the 10-dw
BiodF: Percentage of biodegradation at day 28

When the10-dw began after day 18, the result at D28 was used as the result for the end of the 10-dw.

A coefficient was also calculated for the direct addition method (*C*_DA_) and was used as a reference value for each test. In order to compare the BIM results with the direct addition results, we defined a BIM classification index (*R*_BIM_) as the ratio *C*_BIM_/*C*_DA median_. *C*_BIM_ and *C*_DA_ were determined from the median biodegradation values obtained with each BIM and direct addition. The different *C*_BIM_/*C*_DA_ median ratios were statistically compared to the direct addition ratio RDACDA/CDA median = 1. Three BIM classes were defined as follows:

- Class 1: favorable BIM, with *R*_BIM_ > 1 + MAD of *R*_DA_
- Class 2: neutral BIM, with 1 − MAD of *R*_DA_ ≤ *R*_BIM_ ≤1 + MAD of *R*_DA_
- Class 3: unfavorable BIM, with *R*_BIM_ < 1 − MAD of *R*_DA_

## Results

### Screening of the BIMs for the two substances: level 1

#### Anthraquinone

**Fig. 2:**
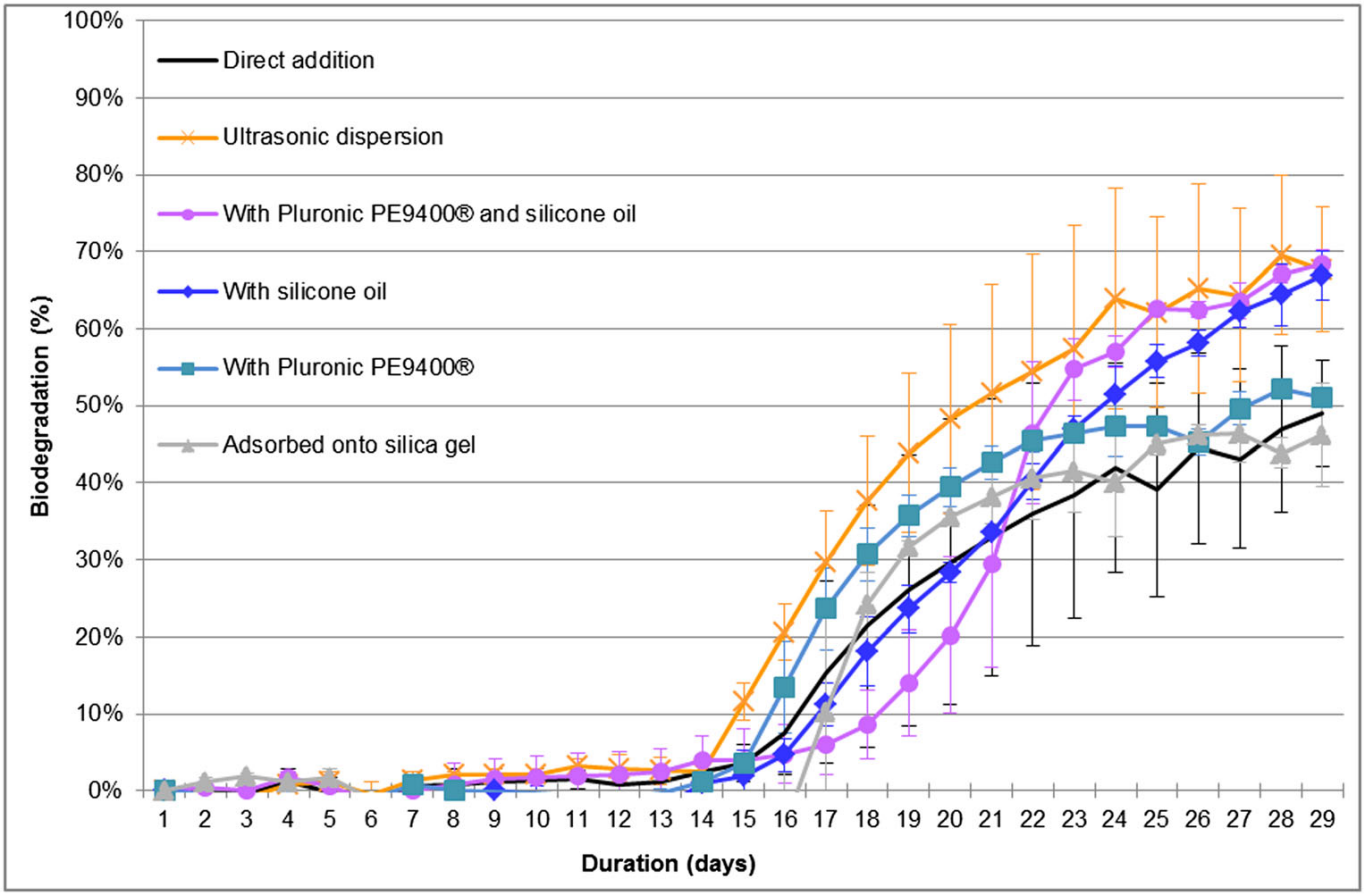
Anthraquinone biodegradation screening test results. Anthraquinone at 20 mg carbon L^−1^ and inoculum at 30 mg dry matter L^−1^. *Curves* represent the median value of five replicates per test condition. *Error bars* represent the median absolute deviation. Operating conditions with anthraquinone: ultrasonic dispersion for 10 min at 35 kHz, silica gel at 5 g L^−1^, dispersed with 10 mL silicone oil L^−1^, dispersed with 10 mg Pluronic 9400® L^−1^, and dispersed with 10 mg Pluronic 9400® L^−1^ and 10 mL silicone oil L^−1^

The biodegradation of anthraquinone without BIM gave a classical sigmoid curve reaching an ultimate biodegradation percentage of 49 % (Fig. 2). Ultrasonic dispersion, dispersion with silicone oil, and emulsion with an emulsifier (Pluronic 9400®) and silicone oil resulted in an improved final biodegradation rate without necessarily accelerating the start of biodegradation. Emulsion with the emulsifier Pluronic PE9400® did not improve the final biodegradation result. When using acetone to adsorb anthraquinone onto silica gel, the residual carbon content due to non-evaporated solvent affected the test result. Moreover, the final result obtained with this BIM showed no improvement in the biodegradation of anthraquinone. The two BIMs “emulsion with Pluronic PE9400®” and “adsorption onto silica gel” also gave similar biodegradation curves compared to the direct addition method.

#### Isodecyl neopentanoate

**Fig. 3:**
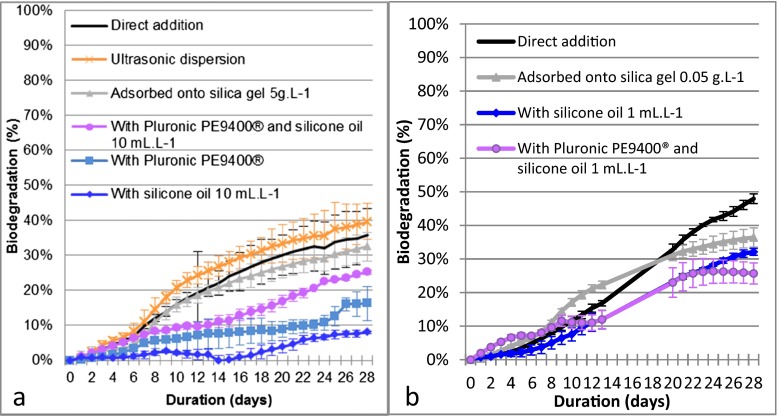
Isodecyl neopentanoate: first screening biodegradation test. Isodecyl neopentanoate at 20 mg carbon L^−1^ and inoculum at 30 mg dry matter L^−1^. *Curves* represent the median value of five replicates per test condition. *Error bars* represent the median absolute deviation. **a** Operating conditions with isodecyl neopentanoate: ultrasonic dispersion for 10 min at 35 kHz, adsorbed onto silica gel at 5 g L^−1^, dispersed with 10 mL silicone oil L^−1^, dispersed with 10 mg Pluronic 9400® L^−1^, and dispersed with 10 mg Pluronic 9400® L^−1^ and 10 mL silicone oil L^−1^. **b** Operating conditions with isodecyl neopentanoate: adsorbed onto silica gel at 0.05 g L^−1^, dispersed with 10 mL silicone oil L^−1^, and dispersed with 10 mg Pluronic 9400® L^−1^ and 10 mL silicone oil L^−1^. CO_2_ consumption was not recorded for days 14 to 19

Biodegradation of isodecyl neopentanoate under direct addition conditions gave a linear profile and reached a final biodegradation percentage of 36 % (Fig. 3a). Ultrasonic dispersion gave a slightly better final biodegradation result of 40 %. Other BIMs (Fig. 3a) failed to provide evidence of improved final biodegradability results. Surprisingly, dispersion with Pluronic 9400® gave lower biodegradation results (16 %) than the direct addition method. Likewise, a high concentration of 10 mL silicone oil L^−1^ gave very low biodegradation at D28 (8 %). In spite of this, the combination of Pluronic 9400® and silicone oil at 10 mL L^−1^ gave a more favorable final result (26 %) than Pluronic 9400® and silicone oil at 10 mL L^−1^ tested separately, although this was less favorable than the final result by direct addition of isodecyl neopentanoate.

Based on these results, it seemed worthwhile to verify the impact of the concentration of oil and silica gel added in the test vessels. Therefore, a second screening test was performed (Fig. 3b) with lower additive concentrations for adsorption onto silica gel, dispersion with silicone oil, and dispersion with Pluronic PE9400® and silicone oil. In this second test, the biodegradation of isodecyl neopentanoate under direct addition conditions reached a final percentage of 48 % (Fig. 3b) as compared to 36 % for the first test, although the 10-dw began 2 days later. The addition of 1 mL L^−1^ of silicone oil led to a more favorable final result (33 %) than with 10 mL L^−1^ (8 %). For adsorption onto silica gel at 0.05 g L^−1^, the final biodegradation result was slightly better than at 5 g L^−1^ (36 and 33 %, respectively). For dispersion with Pluronic PE9400® and silicone oil, the 1 and 10 mL L^−1^ concentrations of silicone oil gave the same final result (25 %).

#### Comparison

These experiments revealed three class 1 BIMs improving the biodegradability of anthraquinone, i.e., ultrasonic dispersion, dispersion with silicone oil, and dispersion with Pluronic PE9400® and silicone oil. The potential of these three BIMs had to be confirmed by standard tests. Two class 2 BIMs were identified with anthraquinone, i.e., adsorption onto silica gel and dispersion with Pluronic 9400®.

For isodecyl neopentanoate, no class 1 BIM was found. In the absence of a favorable BIM, the two class 2 BIMs identified in the screening tests (ultrasonic dispersion and adsorption onto silica gel) were assessed using standard tests. All dispersions with silicone oil, Pluronic PE9400®, and with Pluronic PE9400® and silicone oil were class 3 BIMs.

### Confirmation of BIMs of interest by standard tests: level 2

**Fig. 4:**
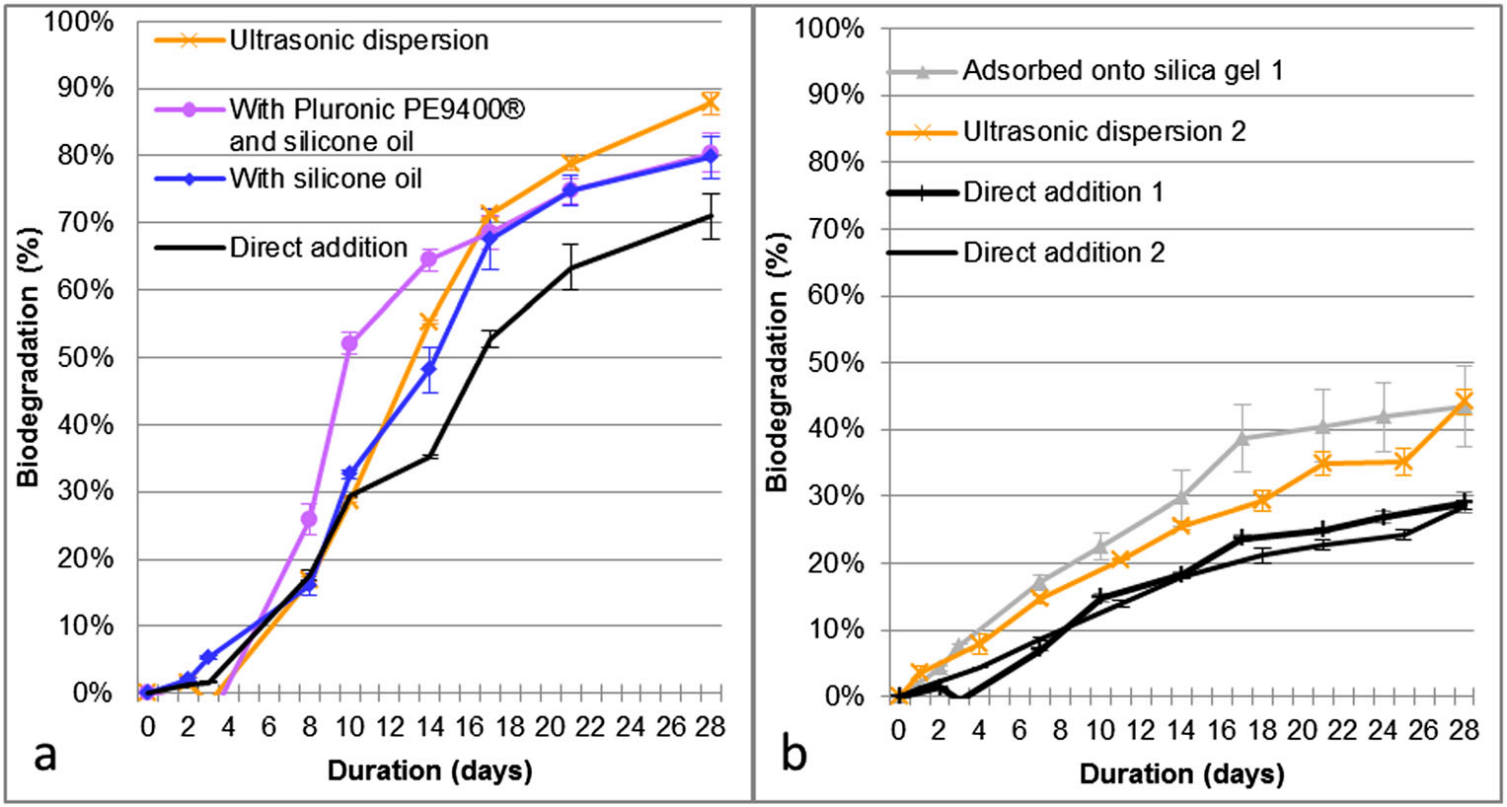
Standard biodegradation tests. *Curves* represent the median value of two replicates per test condition. *Error bars* represent the median absolute deviation. **a** Operating conditions with anthraquinone: direct addition, ultrasonic dispersion for 10 min at 35 kHz, dispersion with silicone oil at 10 mL L^**−**1^, and dispersion with 10 mg Pluronic 9400® L^**−**1^ and 10 mL silicone oil L^**−**1^. **b** Operating conditions with isodecyl neopentanoate: direct addition, ultrasonic dispersion for 10 min at 35 kHz, and adsorption onto silica gel at 0.05 g L^**−**1^

Ultrasonic dispersion, dispersion with silicone oil, and dispersion with an emulsifier and silicone oil gave final biodegradation levels ≥60 % within the 10-dw and improved biodegradation in standard tests for anthraquinone (Fig. 4a). These results confirmed those from the screening tests.

For technical reasons, the two selected BIMs for isodecyl neopentanoate (adsorption onto silica gel and ultrasonic dispersion) could not be performed simultaneously in the same test. These BIMs were therefore assessed in two different tests (tests 1 and 2). However, direct addition of the test substance gave a biodegradation curve showing the same lag phase and linear profile for both tests (Fig. 4b). This biodegradation profile was also similar to that resulting from direct addition in the screening tests. These two BIMs improved the biodegradation of isodecyl neopentanoate and were confirmed as favorable BIMs.

Based upon these experiments, ultrasonic dispersion, dispersion with silicone oil, or dispersion with an emulsifier and silicone oil improved the biodegradation levels of anthraquinone in standard tests and confirmed the results of screening tests. Ultrasonic dispersion and adsorption onto silica gel improved the biodegradation results of isodecyl neopentanoate in the standard tests, allowing it to rise from borderline class 2 (neutral BIM) to class 1 (favorable BIM).

## Discussion

Respicond VI® allows conclusions to be drawn as to the benefits of a BIM in screening ultimate biodegradation tests, especially when the result is clearly favorable or unfavorable for the test substance. The test volume of Respicond® is relatively low (150 mL) but remains compatible with the majority of biodegradation tests (Ingerslev et al. 2000). This test volume may lead to an underestimation of the results due to the lower representativeness of bacterial inoculum sampling, with 4.425 mg of dry matter for 150 mL as compared to 29.5 mg for a test volume of 1 L (Blok and Booy 1984).

The standard ready biodegradability tests confirmed the class 1 (favorable) status of the anthraquinone BIMs. For isodecyl neopentanoate, the two BIMs, previously defined as class 2 (neutral) by the screening test, were found to be class 1 (favorable) in the standard ready biodegradabilty tests, as there was an improvement in biodegradation.

Anthraquinone obtains the status of “readily biodegradable,” but no BIMs allow this status to be confirmed for isodecyl neopentanoate. Isodecyl neopentanoate is an ester that can potentially undergo rapid cleavage, by means of abiotic and biotic mechanisms, into alcohol and an acid. In the absence of analytical monitoring, it is very difficult to conclude whether or not ultimate degradation is actually achieved. The curves obtained do not appear to reach true plateaus after 28 days of incubation. Further tests could be performed over a longer period of time in order to verify the biodegradation potential of isodecyl neopentanoate.

In our evaluation strategy, screening tests could therefore be used as a first-tier test to select class 1 (favorable) BIMs and/or exclude class 3 (unfavorable) BIMs (Table 2).

**Table 2:** Global test strategy for tier 1 and tier 2 results (A: results for anthraquinone, B: results for isodecyl neopentanoate)

BIM		Tier 1—screening test		Tier 2—standard test	
		*R*_BIM_	Class	*R*_BIM_	Class
A	Ultrasonic dispersion	1.29	1	1.18	1
	Silica gel	1.02	2	–	
	Silicone oil 10 mL L^−1^	1.19	1	1.11	1
	Pluronic PE9400®	0.99	2	–	
	Pluronic PE9400® and silicone oil	1.28	1	1.19	1
B	Ultrasonic dispersion	1.06	2	1.21	1
	Silica gel 5000 mg L^−1^	0.96	2	–	
	Silica gel 50 mg L^−1^	0.94	2	1.27	1
	Silicone oil 10 mL L^−1^	0.12	3	–	
	Silicone oil 1 mL L^−1^	0.80	3	–	
	Pluronic PE9400®	0.45	3	–	
	Pluronic PE9400® and silicone oil 10 mL L^−1^	0.75	3	–	
	Pluronic PE9400® and silicone oil 1 mL L^−1^	0.76	3	–	

Class 1: favorable BIM, class 2: neutral BIM, class 3: unfavorable BIM

Since it takes into account three parameters—the start of biodegradation and the percentages of biodegradation at the end of the 10-dw and at the end of the test—the *R*_BIM_ ratio was relevant for defining the impact of BIMs upon biodegradation. Comparison with the reference condition—direct addition—in the same series of experiments made it possible to overcome the variability of the inoculum. The *R*_BIM_ ratio proved to be a good way to compare the impact of sample preparation methods on biodegradation results.

Poorly water-soluble ionic substances involve interactions depending on pH (ionization forms) and salts (solubility), but we cannot rule out potential interference for hydrolysis products in the case of the liquid model compound. For this study, we focused on non-ionic substances to avoid these interactions. Ultrasonic dispersion is an interesting BIM for solid and liquid substances. Unlike the results obtained by Nyholm 1990, ultrasonic dispersion gave better results than the direct addition method for anthraquinone (*R*_BIM_ = 1.18) and for isodecyl neopentanoate (*R*_BIM_ = 1.21).

The silica gel BIM requires a solvent for the solid chemical to being adsorbed onto silica particles. Identifying the appropriate solvent for each test substance is tedious because this solvent should be either completely removed or not readily biodegradable and non-toxic to bacteria and must not be harmful to human health. When acetone was used to adsorb anthraquinone onto the silica gel, the residual carbon content due to the un-evaporated solvent affected the test results. The presence of the solvent led to a high variability of results for this BIM (De Morsier et al. 1987; Nyholm 1990 and Handley et al. 2002). The amount of silica gel added to the test solution depends on the adsorption potential of the substance to the gel and cannot be standardized. This method, associated with the use of a biodegradable solvent (acetone), does not seem appropriate to the evaluation of the ready biodegradability of a solid substance. For liquid chemicals, the amount of silica gel must be defined. It would appear worthwhile to work on adsorbing a hydrophobic substance to silica gel at various rates and evaluating the two sizes of silica gel (15 and 200–500 μm) proposed in the ISO Test Guideline (ISO 1995). This support is inert, is non-toxic to bacteria, and does not constitute a carbon source for the biodegradation test. This method is easily usable for liquid substances, and the standard biodegradability test result obtained for isodecyl neopentanoate with a *R*_BIM_ = 1.27 makes this an attractive option.

The concentration of emulsifier was chosen according to ISO guideline (ISO 1995). The tests performed did not aim to establish the optimal concentration at which the test substance can be properly dispersed without the formation of micelles. A high emulsifier concentration appears to reduce the availability of the test substance to the bacteria. This phenomenon was highlighted in the biodegradability tests with the liquid substance, the results obtained with the emulsifier being worse than those obtained by direct addition. This negative effect was probably due to the limited bioavailability of the substrate trapped in surfactant micelles (Rodrigues et al. 2013). In addition, micelles could have reduced oxygen diffusion that might have impaired bacterial respiration. Increased and decreased biodegradation rates in the presence of surfactants were reported by Volkering et al. 1997. The emulsifier should preferably be non-biodegradable, e.g., Pluronic 9400® (data specified in the supplementary material). If it is biodegradable, an additional control must be included and the presence of the biodegradable emulsifier should not jeopardize the accuracy of the test. The emulsifier must be non-toxic to bacteria, appropriate to the test substance, and added at an optimized concentration. It would therefore appear difficult to apply this BIM to a large number of substances.

Silicone oil AR20® (polydimethylsiloxane) is promising because of its affinity for hydrophobic substances while being non-biodegradable and non-toxic (data specified in the supplementary material). A promising screening test result—with *R*_BIM_ = 1.19 for the solid chemical anthraquinone—was confirmed by the standard ready biodegradability test results, with *R*_BIM_ = 1.11. For the liquid chemical isodecyl neopentanoate, the results for silicone oil were better results at the lowest of the two concentrations tested (1 and 10 mL L^−1^). This result could be attributed to the exceedingly high concentration of silicone oil, which may have trapped the test substance, thereby reducing its availability to the bacteria. When the BIM consisted of mixing with silicone oil and an emulsifier, biodegradation results for the solid substance were significantly improved (*R*_BIM_ of 1.28 and confirmed at 1.19). The formation of surfactant micelles due to a high emulsifier concentration probably reduced bioavailability. Emulsifiers still have some drawbacks such as specific critical micellar concentration (CMC) and specific affinities for each chemical. Optimizing the emulsifier concentration for this BIM could be useful if the performance of the test is significantly better than with silicone oil alone.

BIM optimization requires adjusted concentrations for each test substance while taking into account the emulsifier CMC. Other adjuvants could also be used, such as 2,2,4,4,6,8,8-heptamethylnonane (Auffret et al. 2009), which has interesting characteristics, i.e., non-biodegradable, non-toxic to bacteria, and good solubilizing capacity.

Ultrasonic dispersion improves the biodegradability results of solid chemicals and could be combined with other supports. For example, combination with silicone oil or silica gel could be of interest for both solid and liquid substances.

## Conclusion and perspectives

The use, under defined operating conditions, of a high-throughput screening respirometer, which has been proven capable of producing robust results (Sweetlove et al. 2013), made it possible to reliably compare up to nine different BIMs in a single run. The categorization of BIMs into three classes allowed BIMs to be selected for improving the rate of biodegradation of poorly water-soluble chemicals in ready biodegradability tests.

**Fig. 5:**
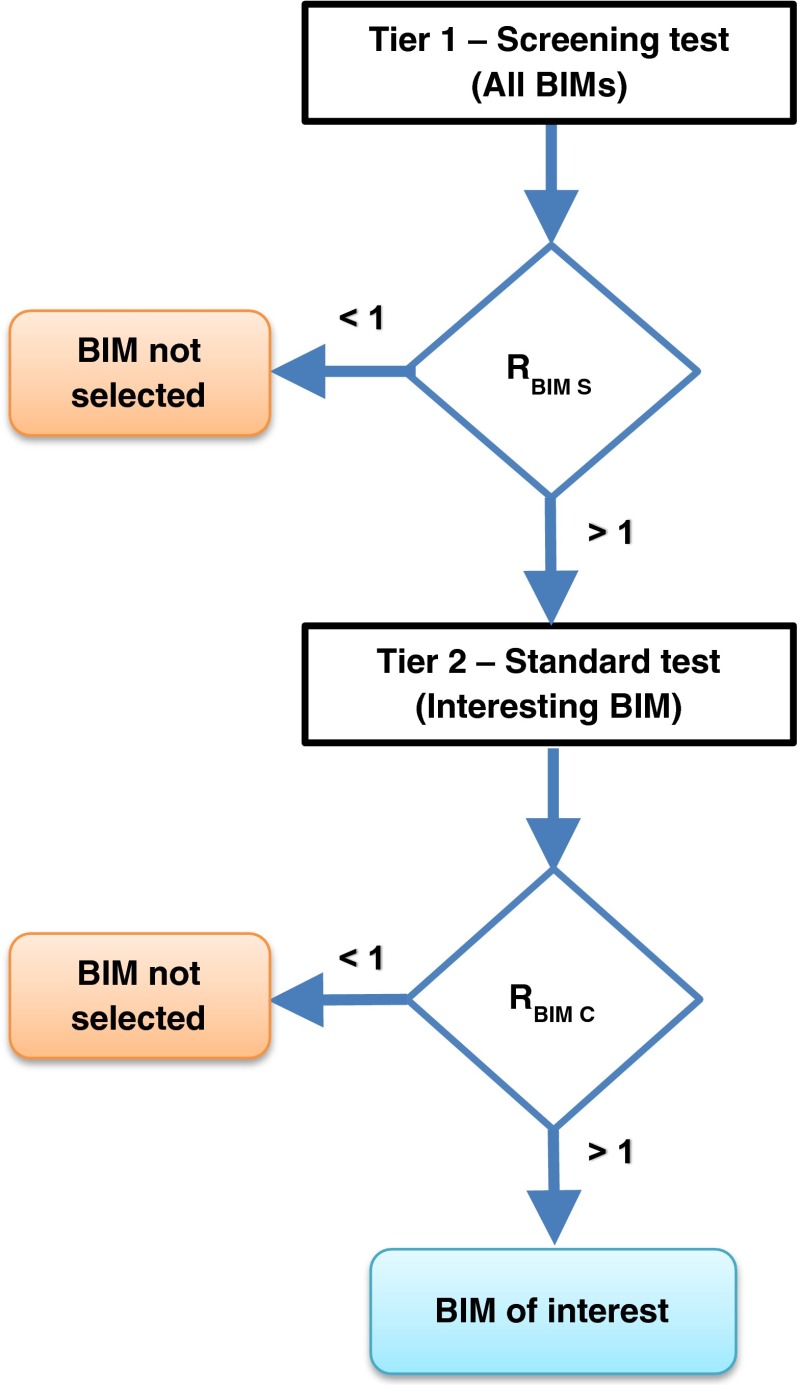
Test methodology. *R*_*BIM S*_ screening BIM, *R*_*BIM C*_ confirmatory BIM

An evaluation strategy was developed for choosing the most appropriate BIM(s) for the assessment of the ready biodegradability potential of poorly water-soluble chemicals in standard tests (Fig. 5).

The *R*_BIM_ ratio makes it possible to compare the different test substance preparation methods and operating conditions used in the various tests. Since this ratio takes into account both the biodegradation curve and the results of direct addition, it is therefore possible to compare and contrast the results of the different tests. This makes it easier to assess the impact of the BIMs on the chemical’s biodegradation.

BIMs of interest differ for the solid and the liquid reference substances used in this study. Enhanced biodegradation results were obtained for the solid substance anthraquinone with the following BIMs: ultrasonic dispersion, silicone oil, and the combination of an emulsifier and silicone oil. In contrast, the results for the liquid substance isodecyl neopentanoate were improved by using ultrasonic dispersion and adsorption onto silica gel. The BIMs featured showed anthraquinone to be consistently readily biodegradable. However, none of the tested BIMs enabled isodecyl neopentanoate to meet the criteria for ready biodegradability.

Future work will focus on the optimization of these BIMs and on the comparison with new BIMs. In order to facilitate the comparison between different BIMs, future work will also focus on reducing the variability of the metabolizing capacity of the microbial inoculum. Furthermore, additional chemicals will be evaluated to improve the proposed strategy and consolidate the results obtained for the liquid and solid chemicals.

## Electronic supplementary material

Below is the link to the electronic supplementary material.

ESM 1 (DOCX 1851 kb)

## Glossary

BIM: Bioavailability improvement method
DOC: Dissolved organic carbon is the organic carbon present in solution or that which passes through a 0.45-μm filter or remains in the supernatant after centrifuging at approx. 4000*g* (about 40.000 m s^−2^) for 15 min (OECD 1992)
MAD: The median absolute deviation is a variability indicator, used for median values
FD: First day when 10 % biodegradation is reached
10-dw—ten-day window: The pass values of the biodegradation test (60 % for OECD 301B) have to be reached in a 10-day window and within the 28-day period of the test. The 10-day window begins when the degree of biodegradation has reached 10 % DOC, ThOD, or ThCO_2_ and must end before day 28 of the test (OECD 1992)
*T*_B_: Percentage of the test duration required by the inoculum for the biodegradation process
ThCO_2_: Theoretical carbon dioxide (mg) is the quantity of carbon dioxide calculated to be produced from the known or measured carbon content of the test compound when fully mineralized, also expressed as milligram carbon dioxide evolved per milligram test compound (OECD 1992)
ThOD: Theoretical oxygen demand (mg) is the total amount of oxygen required to oxidize a chemical completely; it is calculated from the molecular formula and is also expressed as milligram oxygen required per milligram test compound (OECD 1992)
Biod10dw: Biodegradation percentage at the end of the 10-dw
BiodF: Biodegradation percentage at day 28 of incubation
*C*_BIM_: Quantification coefficient allowing results obtained with the different BIMs to being compared
*C*_DA_: Quantification coefficient for the direct addition method. Used as a reference value for each test
*R*_BIM_: Ratio *C*_BIM_/*C*_DA median_. BIM classification index relative to direct addition. *R*_BIM S_ screening *BIM*, *R*_BIM C_: confirmatory BIM
*R*_DA_: Ratio *C*_DA_/*C*_DA median_ for direct addition. Calculated for each replicate of the direct addition results
