# Supplementary material for: Evaluating the ready biodegradability of two poorly water-soluble substances: comparative approach of bioavailability improvement methods (BIMs)
Source: Environ Sci Pollut Res Int. 2016 May 28;23(17):17592–602. doi: 10.1007/s11356-016-6899-3 (PMC5010604; doi:10.1007/s11356-016-6899-3)

**Evaluating the ready biodegradability of two poorly water-soluble substances: Comparative approach of bioavailability improvement methods (BIM’s).**

**Supplementary material**

Please find below (table 3, 4, 5 and 6), the detailed values on biodegradation tests performed in this study.

Table 3: Detailed values of Anthraquinone biodegradation screening test


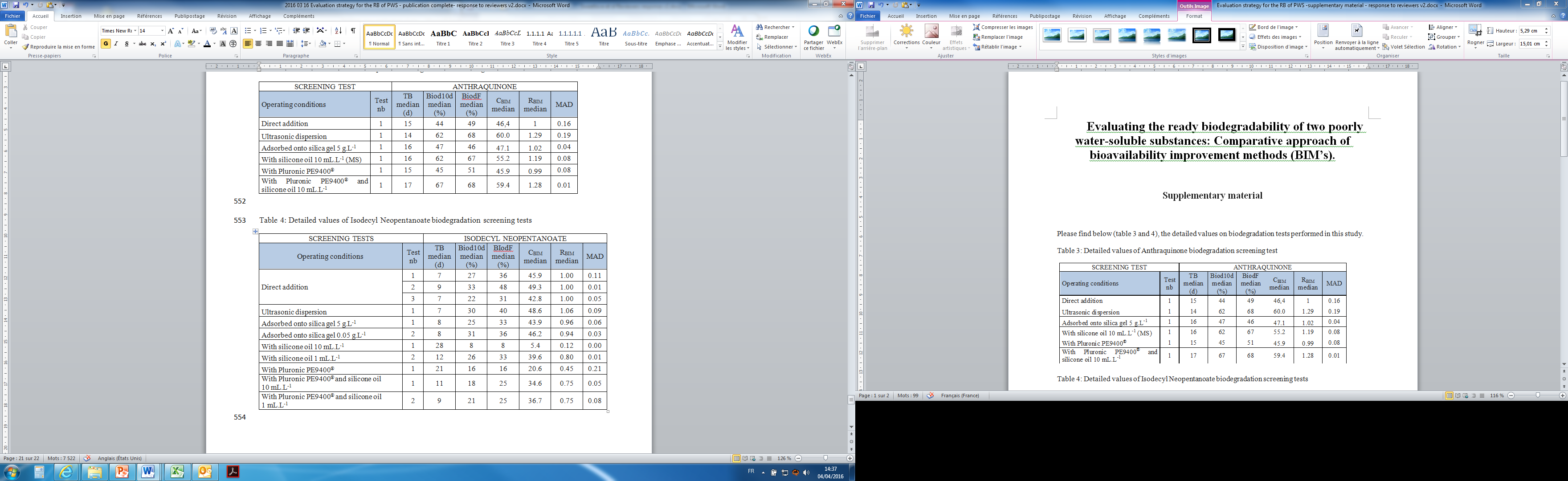


Table 4: Detailed values of Isodecyl Neopentanoate biodegradation screening tests


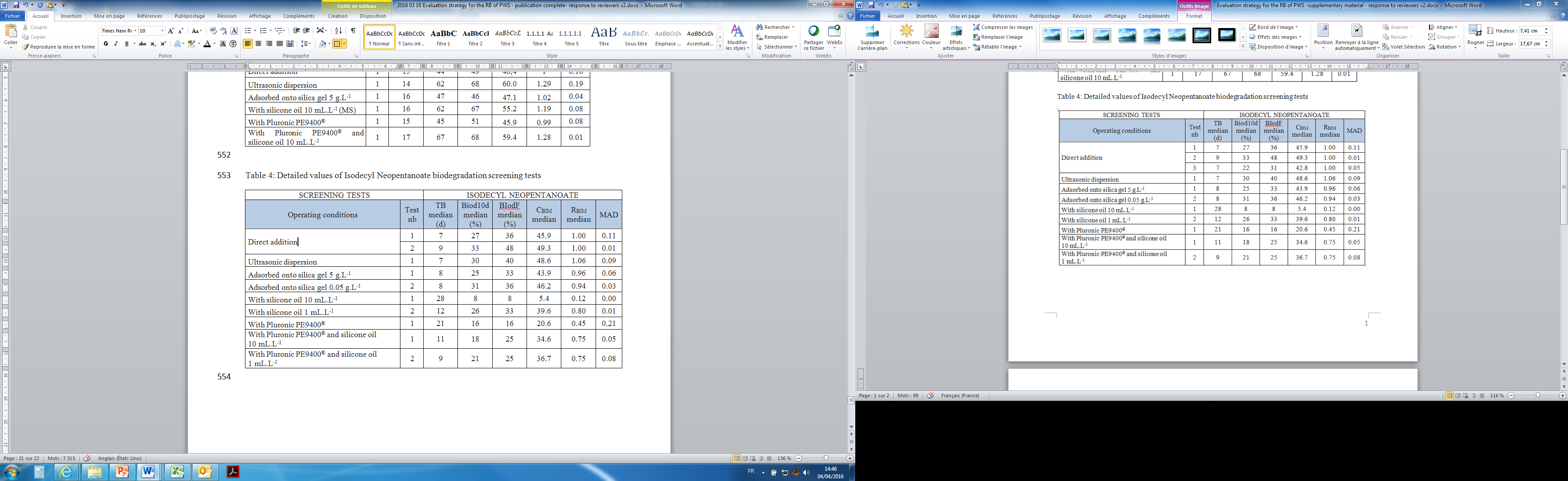


Table 5: Detailed values of Anthraquinone biodegradation standard test


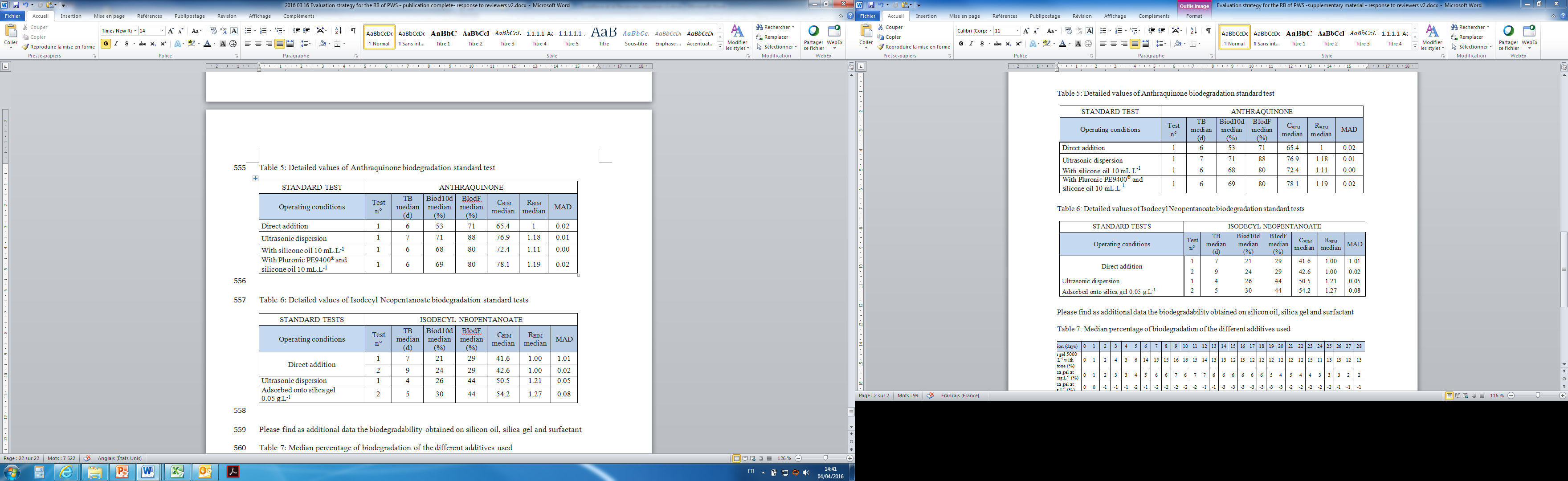


Table 6: Detailed values of Isodecyl Neopentanoate biodegradation standard tests


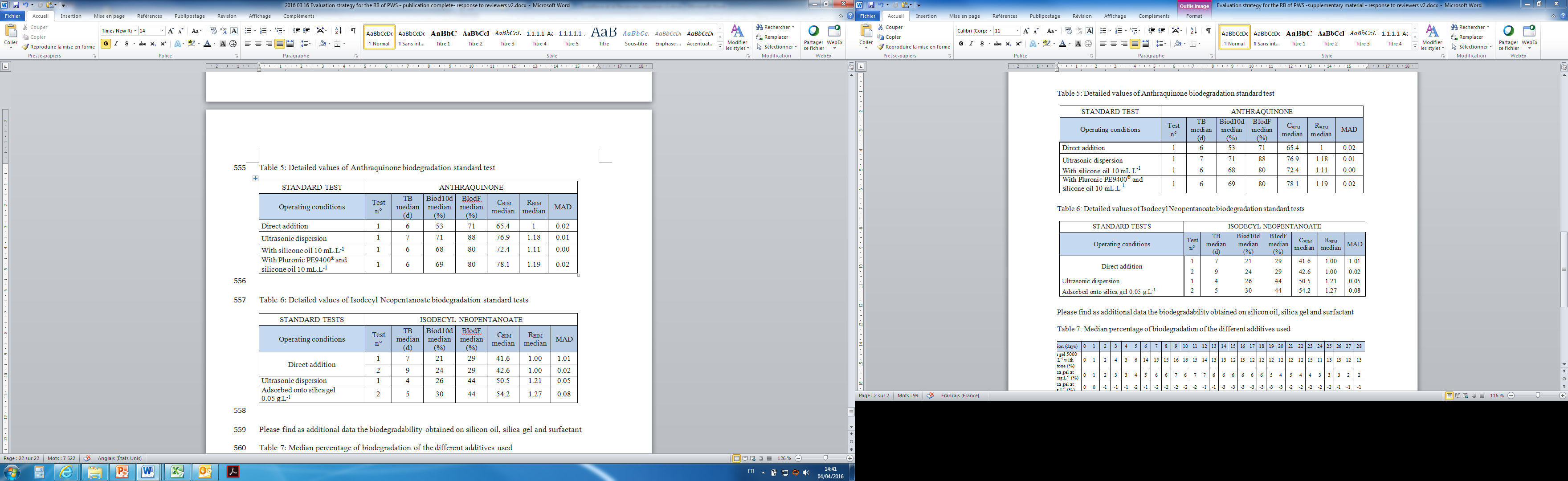


Please find as additional data the biodegradability obtained on silicon oil, silica gel and surfactant (Table 7)

Table 7: Median percentage of biodegradation of the different additives used


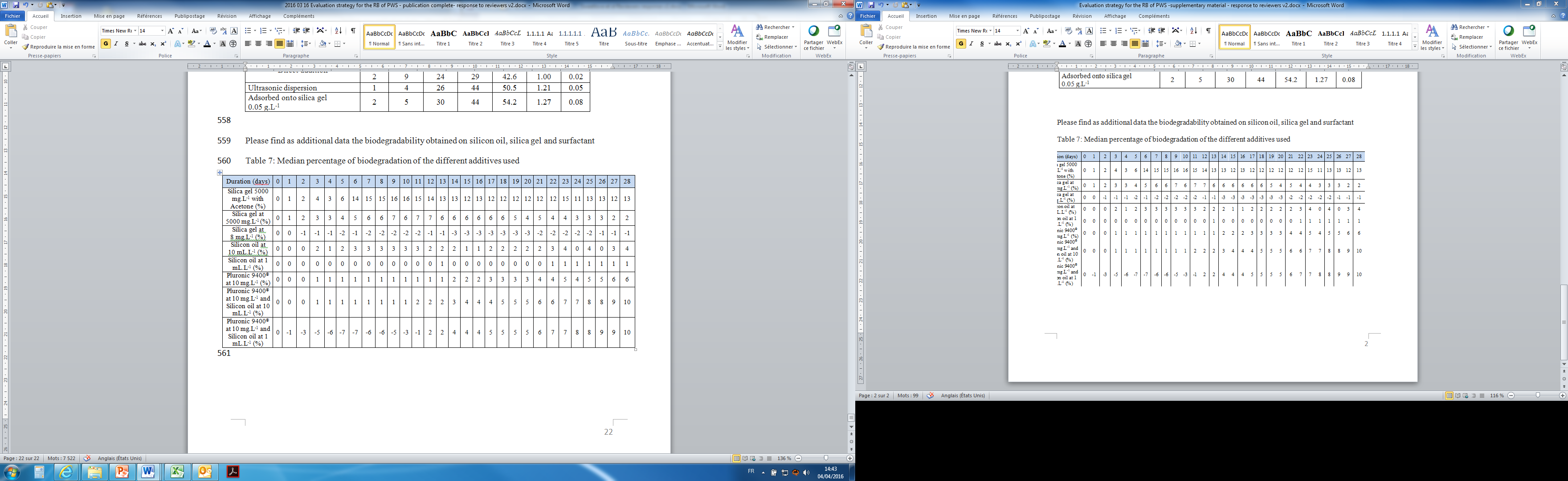

Supplement: Supplementary file 1 — (DOCX 1851 kb) [file 11356_2016_6899_MOESM1_ESM.docx]
